# A bottom-up approach towards a bacterial consortium for the biotechnological conversion of chitin to l-lysine

**DOI:** 10.1007/s00253-021-11112-5

**Published:** 2021-02-01

**Authors:** Marina Vortmann, Anna K. Stumpf, Elvira Sgobba, Mareike E. Dirks-Hofmeister, Martin Krehenbrink, Volker F. Wendisch, Bodo Philipp, Bruno M. Moerschbacher

**Affiliations:** 1 Institute for Biology and Biotechnology of Plants, University of Münster, Schlossplatz 8, 48143 Münster, Germany; 2 Institute for Molecular Microbiology and Biotechnology, University of Münster, Corrensstr. 3, 48149 Münster, Germany; 3 Chair of Genetics of Prokaryotes, Faculty of Biology & CeBiTec, University of Bielefeld, P.O. Box 100131, 33501 Bielefeld, Germany; 4 Present Address: Department of Forest Genetics and Plant Physiology, SLU, Skogsmarksgränd 17, 90183 Umeå, Sweden; 5 WeissBioTech GmbH, An der Hansalinie 48-50, 59387 Ascheberg, Germany; 6 Cysal GmbH, Mendelstraße 11, 48149 Münster, Germany

**Keywords:** Microbial consortia, Chitin, *N*-acetylglucosamine, *Escherichia coli*, *Corynebacterium glutamicum*, Cross-feeding

## Abstract

Chitin is an abundant waste product from shrimp and mushroom industries and as such, an appropriate secondary feedstock for biotechnological processes. However, chitin is a crystalline substrate embedded in complex biological matrices, and, therefore, difficult to utilize, requiring an equally complex chitinolytic machinery. Following a bottom-up approach, we here describe the step-wise development of a mutualistic, non-competitive consortium in which a lysine-auxotrophic *Escherichia coli* substrate converter cleaves the chitin monomer *N*-acetylglucosamine (GlcNAc) into glucosamine (GlcN) and acetate, but uses only acetate while leaving GlcN for growth of the lysine-secreting *Corynebacterium glutamicum* producer strain. We first engineered the substrate converter strain for growth on acetate but not GlcN, and the producer strain for growth on GlcN but not acetate. Growth of the two strains in co-culture in the presence of a mixture of GlcN and acetate was stabilized through lysine cross-feeding. Addition of recombinant chitinase to cleave chitin into GlcNAc_2_, chitin deacetylase to convert GlcNAc_2_ into GlcN_2_ and acetate, and glucosaminidase to cleave GlcN_2_ into GlcN supported growth of the two strains in co-culture in the presence of colloidal chitin as sole carbon source. Substrate converter strains secreting a chitinase or a β-1,4-glucosaminidase degraded chitin to GlcNAc_2_ or GlcN_2_ to GlcN, respectively, but required glucose for growth. In contrast, by cleaving GlcNAc into GlcN and acetate, a chitin deacetylase-expressing substrate converter enabled growth of the producer strain in co-culture with GlcNAc as sole carbon source, providing proof-of-principle for a fully integrated co-culture for the biotechnological utilization of chitin.

Key Points

*• A bacterial consortium was developed to use chitin as feedstock for the bioeconomy.*

*• Substrate converter and producer strain use different chitin hydrolysis products.*

*• Substrate converter and producer strain are mutually dependent on each other.*

**Supplementary Information:**

The online version contains supplementary material available at 10.1007/s00253-021-11112-5.

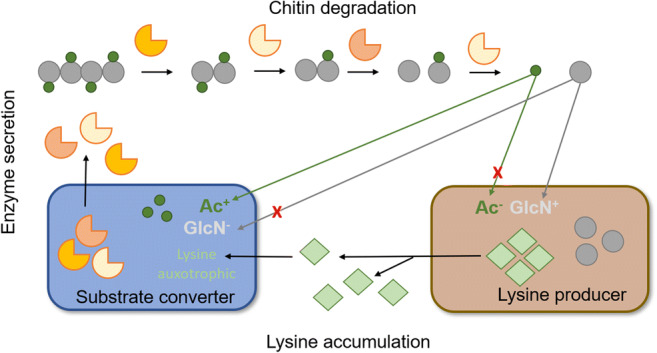

## Introduction

In biotechnology, it is desirable to replace food-grade feedstocks by secondary feedstocks derived from organic waste for both economic and environmental reasons (Wendisch et al. 2016; Abu Yazid et al. 2017). Chitin, one of the most abundant biopolymers on earth, is a polysaccharide that occurs in large amounts in the waste streams of different industries, e.g. in crustacean shells originating from marine fisheries, or fungal mycelium wastes arising from mushroom farming and fungal fermentations for the production of enzymes (Teng et al. 2001; Nisticò 2017). Among these sources, fungal fermentation waste is the most reproducibly available and the least contaminated one (Cai et al. 2006). The volume of this waste stream is difficult to quantify (Ghormade et al. 2017). Acetic acid production by *Aspergillus niger* alone probably yields about 0.1–0.2 Mt of dry mycelium annually, and this may have to be multiplied by a factor of 2–3 when fungal fermentations for other fine chemicals or enzymes are considered as well. Today, this potentially precious resource, in spite of its constant high quality, is most often either burned or transported to landfills instead of being upcycled in the interest of sustainable resource management.

**Fig. 1:**
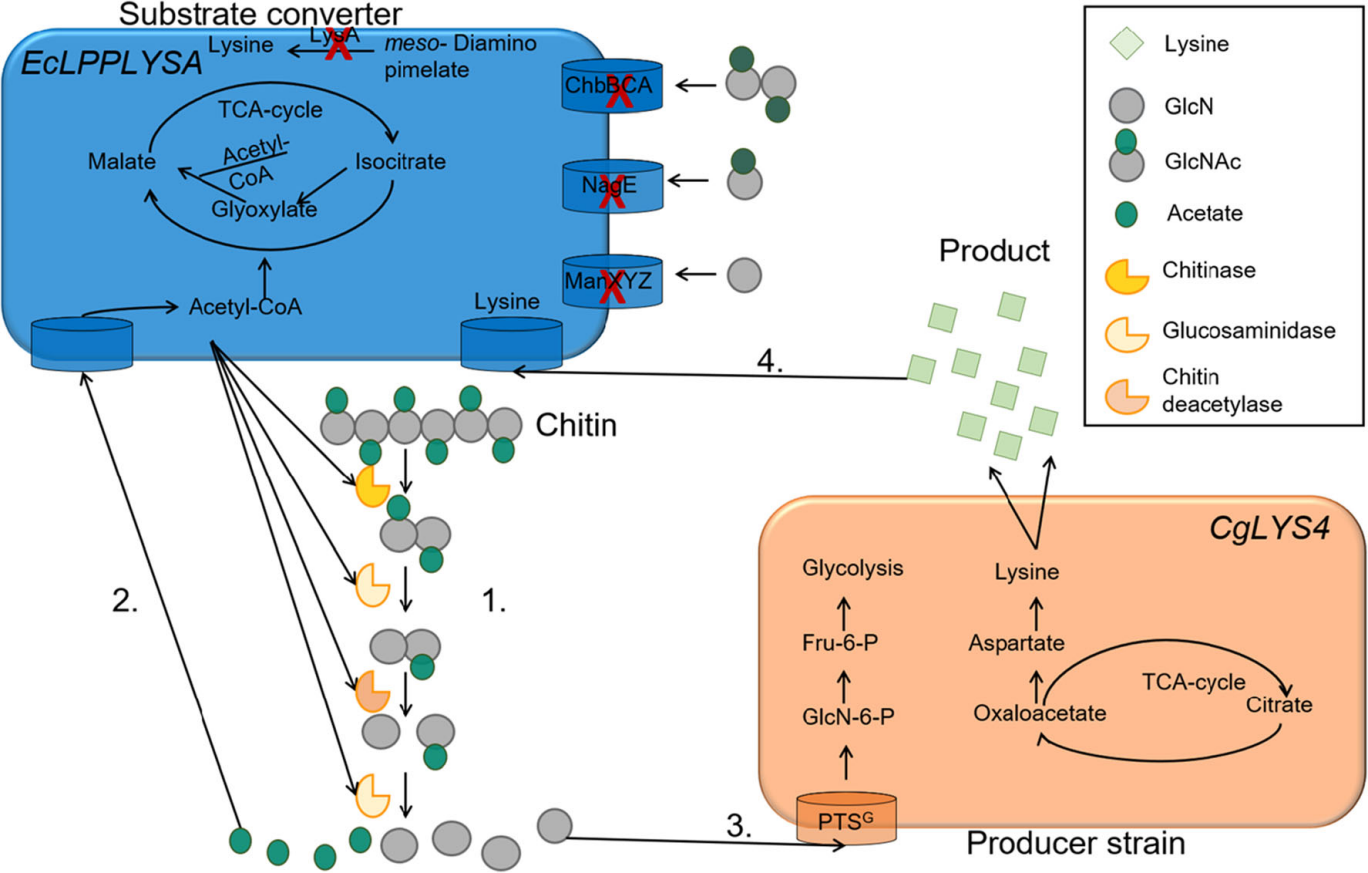
Design of the synthetic mutualistic consortium with *E. coli* and *C. glutamicum* for l-lysine production with chitin as sole source of carbon and energy. (1) The substrate converter EcLPPLYSA (*E. coli* W3110 *ΔnagE ΔmanXYZ ΔchbBCA ΔlysA Δlpp::CM*) expresses heterologous enzymes for the degradation of chitin to glucosamine (GlcN) and acetate. (2) EcLPPLYSA can only use acetate as growth substrate because of deletions in uptake systems for the other chitin degradation products. (3) CgLYS4 (*C. glutamicum* DM1729 *Δpta-ackA Δcat ΔldhA ΔaceAB ΔnanR*) can only use GlcN as growth substrate and for the production of l-lysine because of deletions in acetate metabolism. (4) The consortium is co-stabilized by the lysine auxotrophy of EcLPPLYSA. GlcNAc: *N*-acetylglucosamine, Fru-6-P: fructose-6-phosphate, GlcN-6-P: glucosamine-6-phosphate, LysA: diaminopimelate decarboxylase, ChbBCA: PTS-system chitobiose-specific, NagE: PTS-system N-acetylglucosamine-specific EIICBA component, ManXYZ: mannose-specific PTS-system

Most typical biotechnological producer strains, such as *Corynebacterium glutamicum* or *Escherichia coli,* do not grow on chitin as they lack a functional chitinolytic machinery (Keyhani and Roseman 1997; Verma and Mahadevan 2012). However, expressing chitinolytic enzymes in producer strains has limitations as these strains are usually genetically engineered for maximum product formation so that the additional expression of genes for chitin degradation may lead to decreased productivity (Jagmann and Philipp 2014; Cavaliere et al. 2017). A strategy to overcome this limitation and to uncouple chitin degradation from product formation is the establishment of synthetic microbial consortia (Sgobba and Wendisch 2020). In such a consortium, a substrate converter would generate the carbon and energy source from the substrate, e.g. chitin, for itself and for a producer strain. To avoid competition, the substrate converter should produce two different substrates that are mutually exclusively accessible to the two members of the consortium. The inevitable dependency of the producer strain on the substrate converter can stabilize the consortium, and its robustness can be further increased by implementing an auxotrophy into the substrate converter that is complemented by the producer strain. This concept of co-cultures using alternative feedstocks has mainly been applied for cellulosic substrates (Minty et al. 2013; Wang et al. 2015; Wen et al. 2017) but to our knowledge has only recently been applied for using chitin as a fermentation substrate (Ma et al. 2020). Figure 1 shows a hypothetical chitin-based consortium of a lysine-auxotrophic substrate converter strain which degrades the GlcNAc-polymer chitin to yield GlcN and acetate, growing on the latter product while making the former available for growth and lysine production by a producer strain. In principle, two different approaches could be chosen, either a top-down approach in which an existing consortium would be chosen and optimized to perform as wished, or a bottom-up approach in which the two cooperating strains are build from scratch by adding the required traits one by one (Shin et al. 2010; Gumulya et al. 2018; Gao et al. 2019). We opted for a bottom-up approach, using *E. coli* as a converter and *C. glutamicum* as a producer. In this scenario, the substrate converter heterologously expresses three chitinolytic enzymes: a chitinase, a chitin deacetylase, and a glucosaminidase, but is unable to take up chitobiose, GlcNAc, or GlcN. In contrast, acetate catabolism is disabled in the producer strain, while it can take up GlcN. Following the step-wise bottom-up strategy towards this eventual goal, we initially focused on the carbon sharing of the final degradation products GlcN and acetate between the two members of the consortium. Next, the enzymatic cleavage of GlcNAc into GlcN and acetate by the substrate converter was established by heterologous expression of a suitable chitin deacetylase. The third step is the generation of monomeric GlcNAc from chitin oligomers, and the final fourth step of the bottom-up approach will be the breakdown of the crystalline chitin polymer to GlcNAc oligomers by secretion of a suitable mixture of chitin degrading enzymes.

## Materials and methods

### Bacterial strains and growth experiments

Bacterial strains and plasmids used in this study are listed in appendix Table S1a and S1b. Cells were grown in Luria-Bertani (LB) medium, trypticase soy broth (TSB), modified minimal medium M9 without additional FeCl_2_ (Klebensberger et al. 2006) or minimal medium M9extra (Sgobba et al. 2018). The following carbon and energy sources were used for cultivation in minimal medium: glucose (Glc), sodium acetate, glucosamine (GlcN), *N*-acetylglucosamine (GlcNAc) (all: Sigma Aldrich, München, Germany), *N,N′*-diacetylchitobiose (chitobiose, GlcNAc_2_), de-*N*-acetylated chitobiose (GlcN_2_), or chitin (France Chitine, Orange, France); concentrations of the respective carbon sources are mentioned in the results section. *E. coli* cells used for growth experiments were maintained on solid (1.5% (*w*/*v*) agar) medium M9 using 20 mM Glc as carbon and energy source. For *E. coli* strains harboring plasmids pPRII+ or carrying an integrated chloramphenicol resistance cassette, 17 μg ml^−1^ chloramphenicol (Cm) was added. For maintenance of vector pET-22b (+) in the strain *E. coli* Rosetta 2 (DE3), solid LB medium (1.5% (*w*/*v*) agar) containing 100 μg ml^−1^ ampicillin plus 34 μg ml^−1^ chloramphenicol was used. *C. glutamicum* cells were maintained on solid LB medium containing 50 μg ml^−1^ nalidixic acid. For overnight precultures of *E. coli* strains, 10 ml tubes with 4 ml M9 medium and 20 mM Glc were inoculated from M9 agar plates. Overnight precultures of *C. glutamicum* strains were cultivated in 100 ml Erlenmeyer flasks without baffles containing 10 ml of TSB. All precultures were incubated for 12–14 h. Precultures for all growth experiments were centrifuged for 5 min at 1.800 *x* g, and cells were resuspended in M9 medium without carbon source. Growth was monitored as optical density at 600 nm (OD_600_) with the UV329 mini 1240 spectrophotometer (Shimadzu, Kyōto, Japan) or with the Camspec Visible Spectrophotometer M107 (Spectronic-Camspec Ltd., Leeds, UK). In the case of using chitin as carbon source and in co-cultivation experiments, growth was monitored as colony-forming units (CFU) as previously described by Jagmann et al. (2010) using medium M9 without carbon source for dilution. Growth experiments in single-cultures of *E. coli* strains were generally performed in 10 ml tubes with 4 ml liquid media inoculated from the washed pre-cultures to an OD_600_ of 0.05 or, in case of isopropyl β- d-1-thiogalactopyranoside (IPTG)-induced cells, to an OD_600_ of 0.4. For cultivation of lysine-auxotrophic *E. coli* strains in single-cultures, either 10 mM (precultures) or 2.5 mM (main cultures) l-lysine were added to the medium. Single cultures of *C. glutamicum* strains were performed in 100 ml Erlenmeyer flasks containing 10 ml of the respective medium with an inoculation OD_600_ of 0.1. For co-cultivation experiments, 100 ml Erlenmeyer flasks containing 10 ml of medium M9extra and the respective carbon source were inoculated to an OD_600_ of 0.1 or 0.4 (IPTG-induced cells) for *E. coli* strains and an OD_600_ of 0.1 for *C. glutamicum*. All growth experiments were conducted at 30 °C and 200 rpm in a rotatory shaker (Ecotron, INFORS HT GmbH, Bottmingen, Switzerland) under aerobic conditions, as typically used for industrial amino acid production in *C. glutamicum* (Ikeda and Takeno 2013).

For growth experiments of CgLYS4 with cell-free supernatant of *E. coli* cultures, supernatants of EcLPP* [TkCDA] and a medium blank with 40 mM acetate which was incubated under the same conditions as the EcLPP* [TkCDA] cultures, were processed as described under preparation of cell-free supernatant of *E. coli* strains. As a control for this experiment, freshly prepared, non-processed M9extra medium was used for the culture of CgLYS4. All main-cultures of CgLYS4 were grown with 40 mM GlcNAc as carbon and energy source and the addition of the respective supernatants or fresh medium.

Immediately after inoculation and at several time points thereafter, samples from the cultures were taken to monitor bacterial growth and quantify metabolite concentrations. Samples for metabolite quantification were centrifuged at 18500 x g for 10 min at room temperature. The supernatants were transferred to a new 1.5 ml reaction tube and stored at −20 °C until further use.

### Preparation of colloidal chitin, GlcNAc_2_ and GlcN_2_

Colloidal chitin was prepared as described previously (Jagmann et al. 2010). For preparation of GlcNAc_2_, 75 μg ml^−1^ chitinase ChiB (50 μkat) were incubated with 0.1% (*w*/*v*) of colloidal chitin in citrate buffer (50 mM, pH 6.0) for 3 d at 37 °C and 200 rpm (Ecotron, INFORS HT GmbH, Bottmingen, Switzerland). After three days, 50 μg ml^−1^ chitinase ChiB were added and incubated for two more days. The suspension was centrifuged at 11400 x g for 10 min and the supernatant was transferred to a new reaction tube. The supernatant was lyophilized (Christ BETA 1–16, Martin Christ GmbH, Osterode am Harz, Germany) for 4 d, and the dried pellet was ground and stored at 4 °C until further use. GlcN_2_ was produced by digesting chitosans with varying degrees of acetylation (~1.5%, 10%, and 35%, prepared as described previously by Weikert et al. 2017 (Weikert et al. 2017)) with chitosanase from *Bacillus* sp. MN. GlcN_2_ was separated from other oligomers in the mixture by size exclusion chromatography as described earlier (Weikert et al. 2017) and lyophilized as described above.

### Preparation of cell-free supernatant of *E. coli strains*

To test the activity of extracellular TkCDA in the cell-free supernatant of *E. coli* strains, the strains were cultivated as described above. For producing cell-free supernatants, the respective volume of either a culture harvested in the early stationary phase, or a pre-culture was collected. The culture supernatants were processed by two centrifugation steps at 16100 x g for 15 min at 15 °C followed by filter-sterilization (pore size 0.2 μM). Afterwards, the supernatant was used for cultivation.

### Construction of *E. coli* strains

All primer sequences are shown in the appendix (Tables S2 and S3).

Deletion of genes encoding the proteins NagE (P09323) (Primer 1 and 2), ManXYZ (P69797, P69801, P69805) (Primer 3 and 4), ChbBCA (P69795, P17334, P69791) (Primer 5 and 6), LysA (P00861) (Primer 7 and 8), and Lpp (P69776) (Primer 9 & 10) in the substrate converter *E. coli* W3110 was performed by the method of Datsenko and Wanner (2000). The integration of the chloramphenicol resistance cassette in the respective genes as well as its removal were verified by colony-PCR using primers up- and downstream of the coding region (primers marked with C and the number of the primer used for the deletion e.g. for *nagE*, Primer C1 and C2 were used).

A synthetic operon including the genes encoding the chitinase ChiB (MW376867), the glucosaminidase TK (MW376868), and the chitin deacetylase TkCDA (MW376869) (all genes were optimized for expression in *E. coli*) was synthesized by GenScript (Piscataway, New Jersey, USA). All genes included sequences encoding for N-terminal pelB-leaders and C-terminal StrepII-Tags unless stated otherwise. This operon was ligated into the pPolyRep vector (pPRII+; Grant EP2848691A1) using the *Nde*I and *Hin*dIII restriction sites, yielding pPRII+::Syn_OP. All other vectors used in this study were derived from this vector as described in appendix Table S4. Plasmid sequences were verified via Sanger sequencing.

All PCR reactions were performed using Phusion® High-Fidelity DNA Polymerase (New England Biolabs, Ipswich, MA, USA) according to the manufacturer’s protocol. Vector and insert-fragments were purified using the innuPREP DOUBLEpure kit™ (Analytik Jena, Jena, Germany) according to the manufacturer’s instructions. Vector fragments were digested with *Dpn*I for 30 min at 37 °C to remove template DNA. *Dpn*I was heat-inactivated at 80 °C for 20 min. Standard protocols were used for the heat-shock transformation of *E. coli,* and positive clones were confirmed via colony PCR and sequencing.

### Determination of enzyme activities

To test the activity of enzymes in culture supernatants, EcLPP* [TkCDA], EcLPP* [TK], and EcLPP* [ChiB] were grown in the presence of their respective substrates. The strains were cultivated in 4 ml medium M9extra with 20 mM GlcNAc (EcLPP* [TkCDA]), 20 mM GlcN_2_ and 20 mM Glc (EcLPP* [TK]), or 0.5% (*w*/*v*) colloidal chitin and 20 mM Glc (EcLPP* [ChiB]) at 30 °C and 200 rpm. Culture supernatants were then analyzed by HPTLC as described earlier (Hamer et al. 2014), using GlcN and GlcNAc (Sigma Aldrich, München, Germany) and their oligomers (GlcN_2–6_ and GlcNAc_2–6_, Megazyme, Bray, Ireland) as standards (8 μg of each monomer/oligomer). Degradation products of GlcNAc, GlcN_2_, and colloidal chitin were verified using UHPLC-ELSD-ESI-MS as described previously, and quantified via the ELSD-signal using an external standard curve (Hamer et al. 2015).

### Purification of enzymes

Cultures of 500 ml LB medium containing 17 μg ml^−1^ chloramphenicol were inoculated with 1% (*v*/*v*) of an overnight culture of the respective strain. When the culture reached OD_600_ of approximately 0.8–1, IPTG was added to a concentration of 0.2 mM.

Cells were harvested after 24 h by centrifugation (20 min, 4000 *x* g, 4 °C), resuspended in 20 ml buffer (20 mM trimethylamine (TEA), 400 mM NaCl, pH 8) and frozen at −20 °C for storage. After thawing, cells were treated with 50 U benzonase (Merck Millipore, Burlington, MA, USA) for 20 min at room temperature supplemented with 250 μl of 1 M MgCl_2_ (final concentration 12.3 mM Mg^2+^). Subsequently, cells were lysed by sonication (5 × 10 s, 40% amplitude using the Branson Digital Sonifier Model 250-D (Emerson, Ferguson, MO, USA)) and lysates were centrifuged (40 min, 40,000 x g, 4 °C) to remove insoluble parts from the supernatant.

Enzymes were isolated from the supernatant by FPLC on an ÄKTApure chromatography system (GE Healthcare, Little Chalfont, United Kingdom) using a Streptactin-matrix (1-ml Streptactin superflow plus cartridge, Qiagen, Hilden, Germany). The column was equilibrated with 5 column volumes of washing buffer containing 20 mM TEA in 400 mM NaCl prior to use. After loading of the crude extract onto the column, the column was washed with 10 column volumes of washing buffer. Elution of the enzyme from the column was achieved using 17 column volumes of elution buffer containing 20 mM TEA, 400 mM NaCl, and 2.5 mM desthiobiotin (IBA Life Sciences, Göttingen, Germany). During elution, the UV-signal was monitored, and the fractions with the highest UV-signal were pooled. The eluate was concentrated by ultrafiltration (Vivaspin 20, cut-off 10 kDa, Sartorius AG, Göttingen, Germany).

Protein concentrations were determined with the bicinchoninic-acid based assay (BCA Protein Assay Kit, Pierce) according to the manufacturer’s protocol. The reaction volumes were scaled down to 20 μl of protein sample (diluted 1:10 and 1:50) and 400 μl BCA working solution. Bovine serum albumin (BSA) was used as a standard.

### Determination of acetate concentrations

Acetate concentrations were determined enzymatically using a kit (Essigsäure-kit, R-Biopharm, Darmstadt, Germany). Reagent volumes were scaled down to the following volumes: Solution 1 (TEA-buffer solution, l-malic acid, MgCl_2_ 6 H_2_O): 100 μl; Solution 2 (cofactors ATP, CoA and NAD^+^): 20 μl; H_2_O: 63 μl; Solution 3 (l-malate dehydrogenase and citric acid synthase): 20 μl; Solution 4 (acetyl-CoA-synthase): 20 μl.

### Statistics

Differences between mean values were tested for significance using a *T*-test, preceded by an *F*-test.

### Accession numbers

The sequences of the genes expressed in the substrate converter were optimized for expression in *E. coli* and can be found in GenBank: chitinase ChiB of *Serratia marcescens* (GenBank Accession no. MW376867); glucosaminidase TK of *Thermococcus kodakarensis* KOD1, also referred to as Tk-Glm (GenBank Accession no. MW376868); chitin deacetylase TkCDA of *Thermococcus kodakarensis* KOD1, also referred to as Tk-dac (GenBank Accession no. MW376869).

## Results

### Introducing metabolic deficiencies into substrate converter strain and producer strain

To establish sharing of the carbon sources resulting from chitin degradation, we first had to introduce metabolic deficiencies into the substrate converter and the producer strain.

**Fig. 2:**
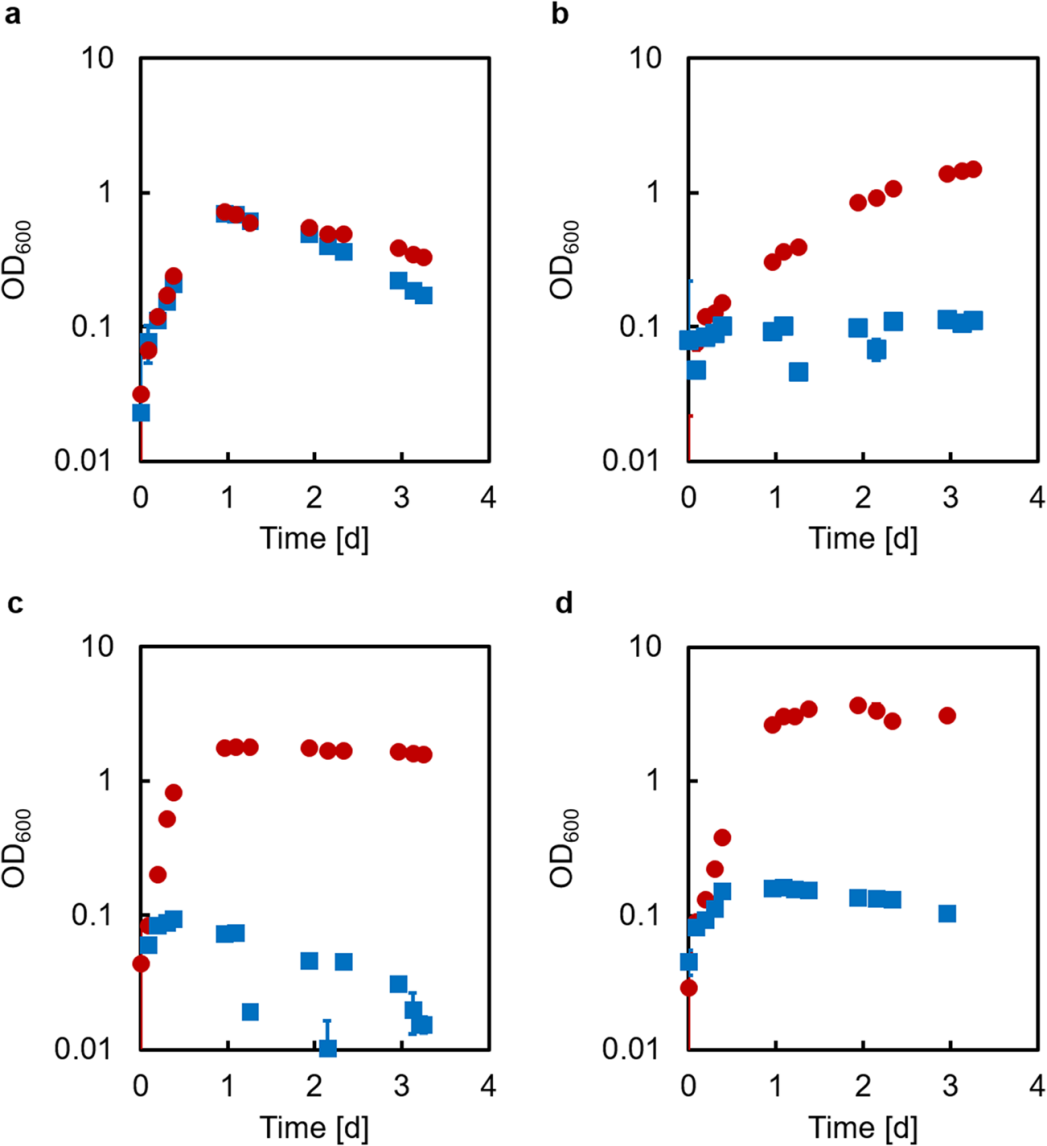
Growth of the *E. coli* wild type strain EcWT (red circles) and its mutant strain EcLPPLYSA* (blue squares) on 20 mM (a) sodium acetate, (b) glucosamine, (c) *N*-acetylglucosamine, or (d) chitobiose. Error bars (mostly smaller than symbols) indicate standard deviation of three independent experiments (*n* = 3)

In the *E. coli* substrate converter strain, genes encoding transporters responsible for the uptake of the amino sugars GlcNAc (*nagE*) and GlcN (*manXYZ*) as well as for the intermediate chitin degradation product chitobiose (Plumbridge and Pellegrini 2004; Verma and Mahadevan 2012) (*chbBCA*) were deleted. In addition, the *lpp* gene, encoding Braun’s lipoprotein, which is located in the outer membrane of *E. coli*, was deleted to improve secretion of the recombinantly expressed enzymes (Shin et al. 2010; Chen et al. 2014; Müller et al. 2016) (Supplemental Fig. S1) which were later introduced for chitin degradation. For simplicity, the *E. coli* substrate converter with the genotype ∆*nagE* ∆*manXYZ* ∆c*hbBCA* ∆*lpp* will be referred to as EcLPP or EcLPP* (‘*’ indicating that the chloramphenicol resistance gene was removed). Furthermore, lysine-auxotrophic variants of EcLPP/EcLPP* were created by deleting the *lysA* gene, and these strains were named EcLPPLYSA/EcLPPLYSA*. EcLPPLYSA* was tested for its ability to grow on acetate, GlcN, GlcNAc, and GlcNAc_2_ compared to the *E. coli* W3110 wildtype strain (EcWT; Fig. 2).

While the wild type strain EcWT was able to grow on all four substrates, the mutant strain EcLPPLYSA* had lost its ability to grow on GlcN, GlcNAc, and GlcNAc_2_ (Fig. 2b–d) while still growing on acetate in the presence of Lys (Fig. 2a).

**Fig. 3:**
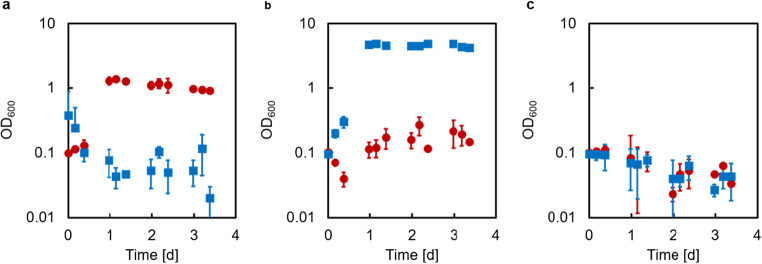
Growth of *C. glutamicum* wild type strain DM1729 (red circles), and its mutant strain CgLYS4 (blue squares) on 20 mM (a) sodium acetate, (b) glucosamine, or (c) *N*-acetyl-glucosamine. Error bars indicate standard deviation of three independent experiments (n = 3)

In the *C. glutamicum* producer strain, the genes encoding acetate kinase (*ackA*), phosphotransacetylase (*pta*), acetyl-CoA:CoA transferase (*cat*), isocitrate lyase (*aceA*), and malate synthase (*aceB*) were deleted to prevent the strain from using acetate (Veit et al. 2009). The *nanR* gene, encoding a repressor of the genes *nagA* (*N*-acetyl-d-glucosamine-6-phosphate deacetylase) and *nagB* (glucosamine-6-phosphate deaminase) was deleted to allow growth on glucosamine (Uhde et al. 2013). Moreover, potential cross-feeding of lactate to the substrate converter was prevented by deletion of *ldhA* (NAD^+^-dependent-l-lactate-dehydrogenase; (Okino et al. 2008)). The resulting strain named CgLYS4 was then tested for growth on acetate, GlcN, and GlcNAc compared to the wildtype *C. glutamicum* (DM1729; Fig. 3).

While the wild type strain DM1729 grew well only on acetate and poorly on glucosamine, the mutant strain CgLYS4 grew well on GlcN, but not on acetate. As expected, both wild type and mutant were unable to grow on GlcNAc, since they lacked the GlcNAc PTS system (NagE) (Matano et al. 2014).

### Simultaneous growth of substrate converter and producer strains on a mixture of acetate and glucosamine

**Fig. 4:**
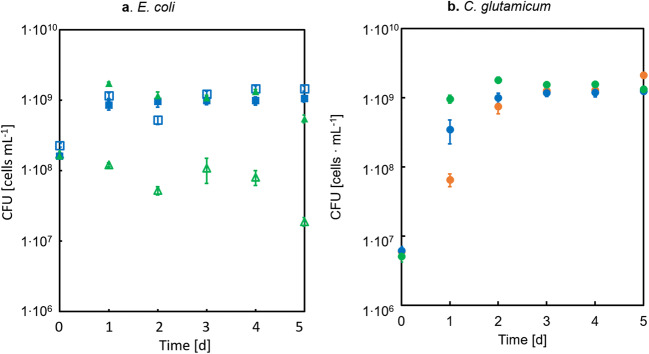
Growth of the *E coli* substrate converter strain EcLPP or EcLPPLYSA and the *C. glutamicum* producer strain CgLYS4 in single and co-cultures with a mixture of 40 mM glucosamine and 40 mM acetate as sole carbon and energy source. (a) CFUs of strain EcLPP (blue squares) and EcLPPLYSA (green triangles) in single culture (open symbols) and in co-culture with CgLYS4 (closed symbols). (b) CFUs of strain CgLYS4 in single culture (orange dots), in co-culture with ECLPP (blue dots) and in co-culture with EcLPPLYSA (green dots). Error bars indicate standard error of the mean of three independent experiments with triplicate determinations each (n = 3)

Next, the *E. coli* substrate converter strain EcLPP or EcLPPLYSA was grown in co-culture with the *C. glutamicum* producer strain CgLYS4 in the presence of their respective substrates, acetate and GlcN, to test whether both strains were able to grow together and whether CgLYS4 can complement the lysine auxotrophy of EcLPPLYSA (Fig. 4).

The producer stain CgLYS4 grew well both in single culture and in co-culture with either of the two converter strains. In contrast, only the substrate converter strain EcLPP was able to grow in single culture as well as in co-culture with CgLYS4, while growth of the lysine-auxotrophic strain EcLPPLYSA required the presence of CgLYS4 providing lysine. These results clearly demonstrated that the strains showed no growth interference and that lysine cross-feeding was successful.

### Testing functionality of the chitin deacetylase secreted by the substrate converter

**Fig. 5:**
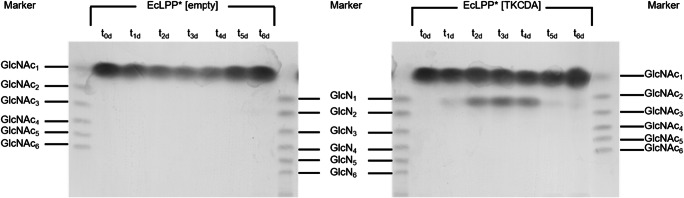
HPTLC analysis of culture supernatants after 0–6 days. Culture supernatant of a culture of EcLPP* [empty] and of a culture of EcLPP* [TkCDA] supplied with 40 mM of GlcNAc, at time points t0d-t6d. Application volumes were 15 μl and 4 μl for samples and standards, respectively. Marker: standard GlcNAc-GlcNAc_6_ or GlcN-GlcN_6_ (each 2 mg ml^−1^). Identification of GlcNAc and GlcNAc_2_ was verified using UHPLC-ESI-MS^n^ (Supplemental Fig. S2)

After engineering the carbon catabolism of the substrate converter strain and the producer strain to grow on the substrates acetate and GlcN, respectively, the substrate converter had to be equipped with genes encoding enzymes for degrading chitin to GlcN and acetate. Following the bottom-up approach, we first introduced, under control of a P*tac* promoter, a chitin deacetylase (CDA) from *Thermococcus kodakarensis* KOD1 (TkCDA) that hydrolyzes GlcNAc to yield GlcN and acetate (Tanaka et al. 2004). As *T. kodakarensis* is a hyperthermophile, we tested the temperature dependency of the enzyme and found maximum enzyme activity at 54 °C, and about one third lower activity at 37 °C (data not shown). The resulting strain EcLPP* [TkCDA] was incubated with GlcNAc and analyzed for growth and conversion of GlcNAc into GlcN. The strain grew with 20 mM GlcNAc to a 2.4-fold higher OD_600_ than the empty vector control (Supplemental Fig. S2a). Shortly prior to the onset of growth, conversion of GlcNAc to GlcN was observed (Fig. 5, Supplemental Fig. S2b), which was not detected in the control strain (Supplemental Fig. S2b), verifying the expression of functional TkCDA in the substrate converter EcLPP* [TkCDA]. Unexpectedly, a decrease of GlcN was observed for strain EcLPP* [TkCDA] after five days, accompanied by a strong increase of OD_600_. Apparently, the substrate converter can still utilize GlcN but not GlcNAc upon prolonged incubation despite the deletion of *manXYZ*.

### Co-culture and lysine cross feeding of substrate converter and producer strains on GlcNAc

**Fig. 6:**
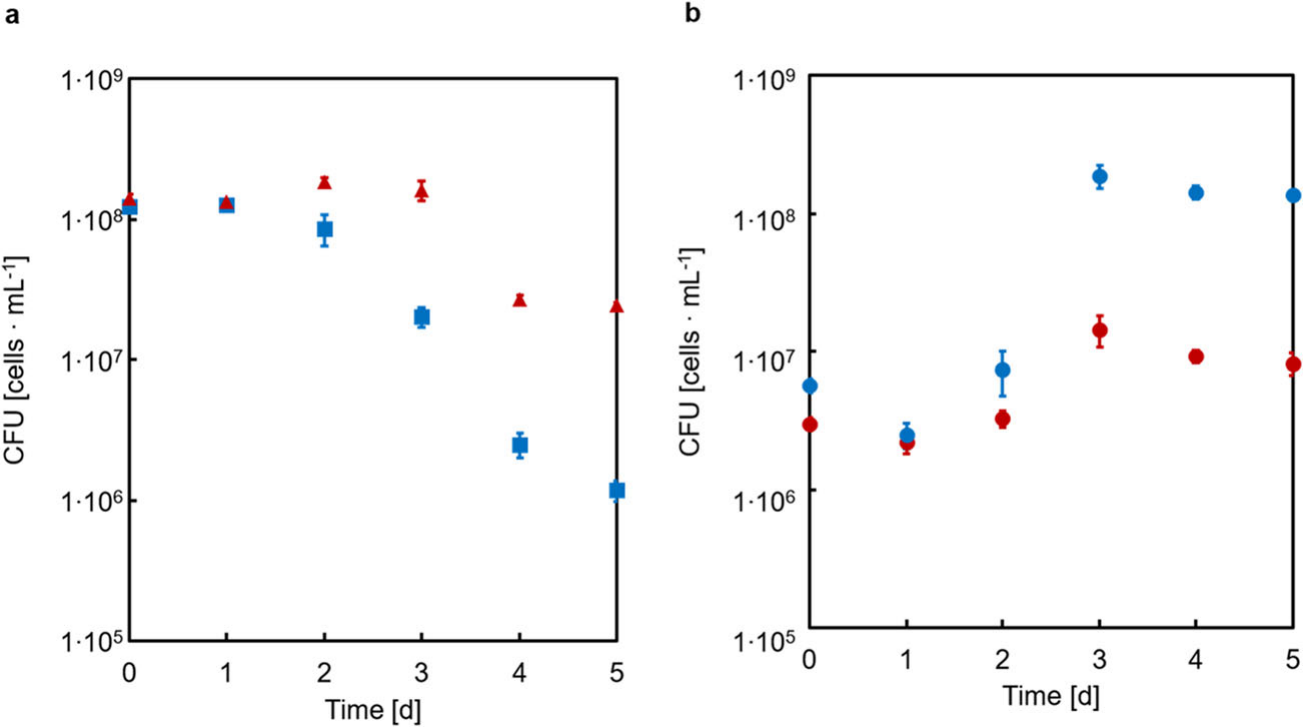
Growth of the *E. coli* substrate converter strain EcLPPLYSA* [TkCDA] expressing a functional chitin deacetylase or the control strain EcLPP* [empty] and of the *C. glutamicum* producer strain CgLYS4 in co-cultures with 40 mM GlcNAc as sole carbon and energy source. CFUs of strain (a) EcLPPLYSA* [TkCDA] (blue squares) or EcLPP* [empty] (red triangles) and (b) CgLYS4 in co-culture with EcLPPLYSA* [TkCDA] (blue dots) and in co-culture with EcLPP* [empty] (red dots). Error bars indicate standard error of the mean of three independent experiments with triplicate determinations each (n = 3)

After demonstrating functional expression of TkCDA in the substrate converter, the next step of the bottom-up approach was to establish the co-culture of the substrate converter EcLPPLYSA* [TkCDA] and the producer strain CgLYS4 using GlcNAc as carbon source (Fig. 6). In this co-culture, a decrease in CFUs of the substrate converter was detected after two days, whereas the producer CgLYS4 showed a distinct increase in CFUs when compared to a co-culture with a control substrate converter harboring an empty vector. Clearly, the heterologous expression of TkCDA in the substrate converter strain EcLPPLYSA* [TkCDA] supported the growth of the producer strain CgLYS4.

**Fig. 7:**
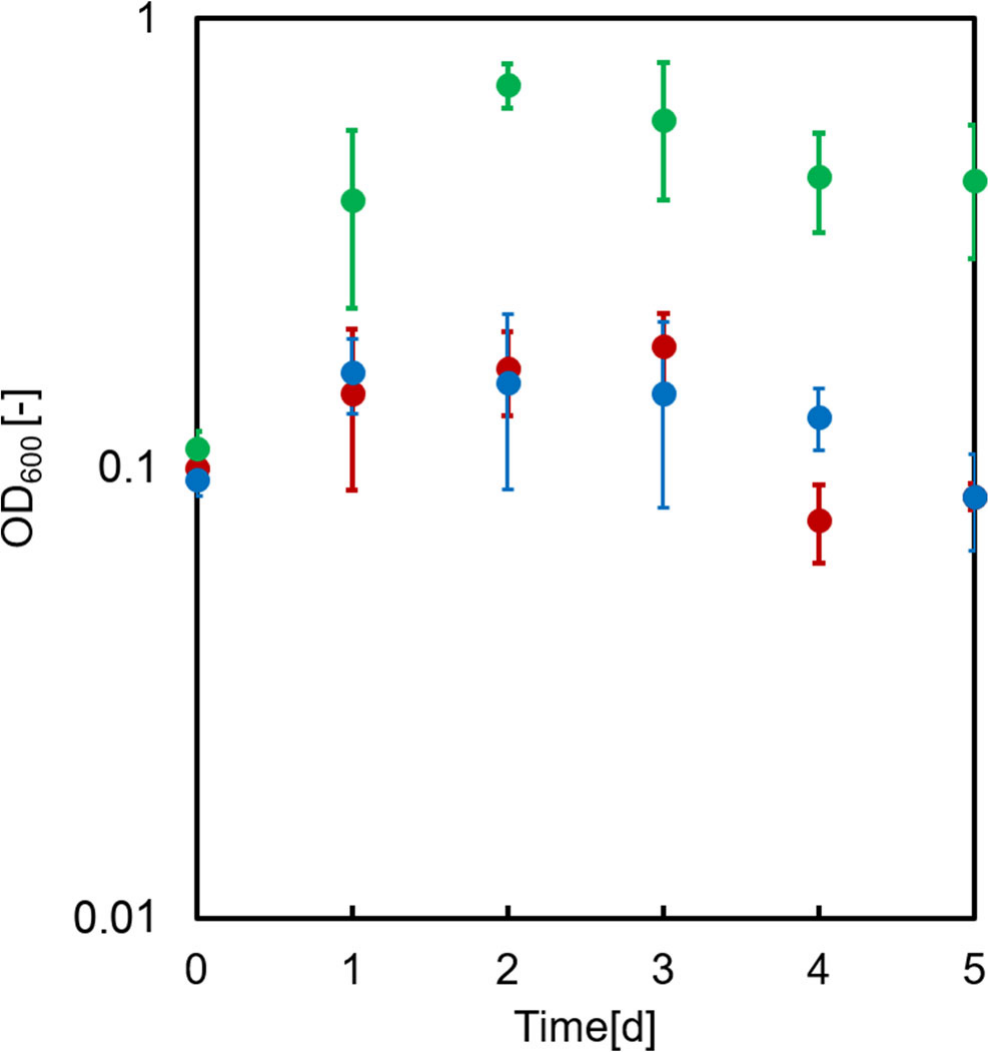
Growth of CgLYS4 with 40 mM GlcNAc as sole carbon and energy source with cell-free culture supernatant of EcLPP* [TkCDA] (green dots) or supernatant of an M9extra medium blank with 40 mM acetate (blue dots) and a control without addition of supernatant (red dots). Error bars indicate standard error of the mean of three independent experiments with triplicate determinations each (n = 3)

To further support this conclusion, two control experiments were performed. First, we investigated whether growth of the producer strain was indeed caused by the extracellular production of GlcN due to secretion of active TkCDA into the medium by the substrate converter strain. To this end, the producer strain CgLYS4 was cultured in M9extra medium with GlcNAc as sole carbon source, supplemented with either cell-free culture supernatant of the substrate converter EcLPP* [TkCDA] or, as a control, fresh culture medium (see Materials and Methods). Strain CgLYS4 only grew in the presence of EcLPP* [TkCDA] culture supernatant, but not in the presence of the control supernatant (Fig. 7).

**Fig. 8:**
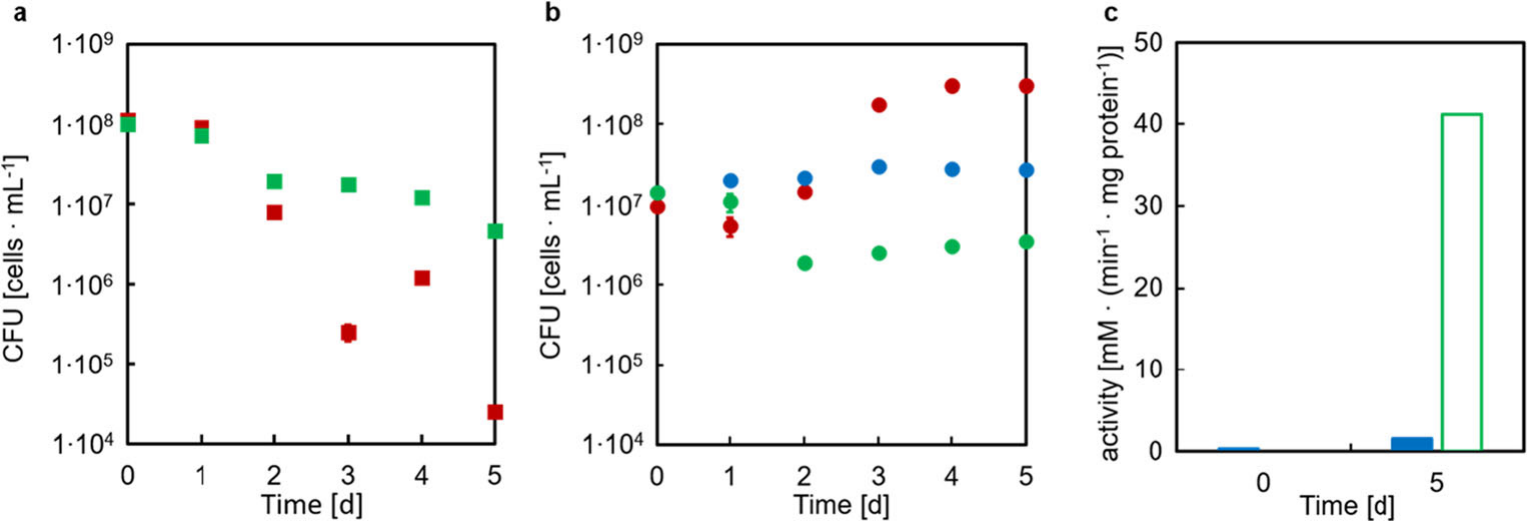
Growth of *E. coli* strain EcLPPLYSA* [TkCDA] and *C. glutamicum* strain CgLYS4 in co-culture with 40 mM GlcNAc as sole carbon and energy source. CFU of strain (a) EcLPPLYSA* [TkCDA] and (b) CgLYS4. Red symbols represent CFU of co-culture with addition of IPTG, green symbols represent CFU without addition of IPTG and blue symbols with addition of culture supernatant of EcLPPLYSA* [TkCDA] cells and no IPTG. Error bars indicate standard error of the mean of three independent experiments with triplicate determinations each (n = 3). (c) Activity of TkCDA of supernatants taken from co-cultures with (green, open bars) and without IPTG (blue, filled bars) at t0d and t5d measured by determination of acetate

The second control experiment was designed to exclude that growth of strain CgLYS4 in the co-culture solely relied on the amount of extracellular TkCDA produced during pre-culture even in the absence of IPTG and transferred to the main culture, rather than by TkCDA secreted into the medium by the substrate converter during co-culture. To this end, three different cultures of CgLYS4 with or without EcLPPLYSA* [TkCDA] were set up: In a first co-culture, expression of TkCDA was induced with IPTG; in a second one, no IPTG was added; and the third culture of CgLYS4 alone was supplied with filter-sterilized supernatant of an EcLPPLYSA* [TkCDA] pre-culture grown on 20 mM Glc and 10 mM lysine that was not induced with IPTG. Strain CgLYS4 only grew in the co-culture with the IPTG-induced EcLPPLYSA* [TkCDA] strain (Fig. 8b). For the co-culture with cell-free supernatant, the CFUs of CgLYS4 remained constant. A decrease of CFUs was observed for the co-culture without addition of IPTG. For the *E. coli* strains, either with or without IPTG, a decrease of the CFU was detected (Fig. 8a). No CFUs of *E. coli* cells were detectable for the filter-sterilized supernatant of an EcLPPLYSA* [TkCDA]. Determination of the TkCDA activity in cell-free supernatants of these co-cultures revealed that TkCDA only increased when IPTG was added to the co-culture (Fig. 8c). In summary, the control experiments showed that growth of the producer strain CgLYS4 in the co-culture depended on the expression and secretion of TkCDA by the substrate converter EcLPPLYSA* [TkCDA] during the co-cultivation.

### Co-culture and lysine cross-feeding of substrate converter and producer strains on colloidal chitin with added or heterologously expressed chitinolytic enzymes

After successfully establishing a mutualistic co-culture with the chitin monomer GlcNAc, we next aimed for the utilization of polymeric chitin by the enzymatic degradation of chitin to GlcNAc. The processive chitinase ChiB of *S. marcescens* (Brurberg et al. 1996; Horn et al. 2006) was chosen to break down polymeric chitin to small chitin oligomers (GlcNAc_n_), mostly chitobiose GlcNAc_2_ with some chitotriose GlcNAc_3_. These chitin oligomers can then be de-*N*-acetylated at their non-reducing ends by the TkCDA yielding acetate and mono-deacetylated chitosan oligomers (GlcN-GlcNAc_(n-1)_). Next, the glucosaminidase TK of *T. kodakarensis* KOD1 (Tanaka et al. 2003) can cleave off the GlcN units at the non-reducing ends, yielding fully acetylated chitin oligomers, each one unit shorter than the original ones (GlcNAc_(n-1)_). Consecutive reciprocal action of TkCDA and TK, thus, completely converts the ChiB products into GlcN monomers and acetate because unlike most CDA, TkCDA can use the monomer GlcNAc as a substrate, cleaving it into GlcN and acetate (Tanaka et al. 2004).

**Fig. 9:**
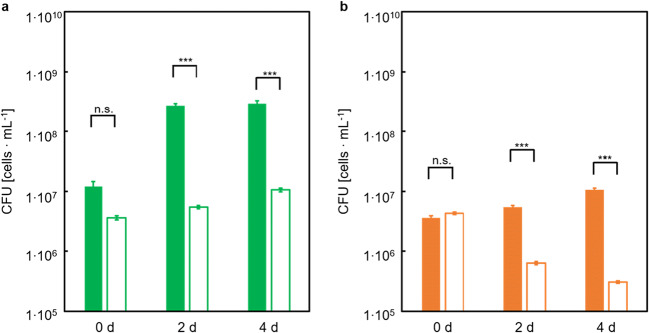
Growth of *E. coli* strain EcCHB and *C. glutamicum* strain CgLYS4 in co-cultures with 0.5% (*w*/*v*) colloidal chitin as sole carbon and energy source. (a) CFUs of strain EcCHB with (filled bars) and without (open bars) addition of enzymes. (b) CFUs of strain CgLYS4 with (filled bars) and without (open bars) addition of enzymes. Addition of purified enzymes: 15 μg ml^−1^ chitinase ChiB, 22.5 μg ml^−1^ TK, and 7.5 μg ml^−1^ TkCDA. Error bars indicate standard error of the mean (*n* = 2); n.s: not significant, *: statistically significant at *P* < 0.05, ** *P* < 0.01, *** *P* < 0.001

To verify whether the combination of these three enzymes – ChiB, TkCDA, and TK – can indeed degrade polymeric chitin to GlcN and acetate and, thus, are suited to be used for the co-culture, a proof-of-principle experiment was performed, in which purified enzymes recombinantly produced in *E. coli* Rosetta 2 (DE3) were added to a co-culture of the substrate converter (genotype ∆*nagE* ∆*manXYZ* ∆c*hbBCA::CM*, named EcCHB, an early version of the converted strain which was later developed into EcLPPLYSA*) and the producer strain CgLYS4 with colloidal chitin as sole carbon source. We resorted to a precursor strain that a) was not lysine-auxotrophic and b) did not carry the *lpp* deletion in order to exclude growth problems due to lysine-auxotrophy and outer membrane instability due to *Δlpp* deletion (Kowata et al. 2016). Without the addition of enzymes, the substrate converter EcCHB showed only minimal growth, while the producer strain CgLYS4 showed a significant decrease in CFUs over a period of four days. In contrast, in the presence of enzymes, the substrate converter showed strong growth and the producer strain grew slightly, indicating that addition of the enzymes did support cell viability (Fig. 9).

**Fig. 10:**
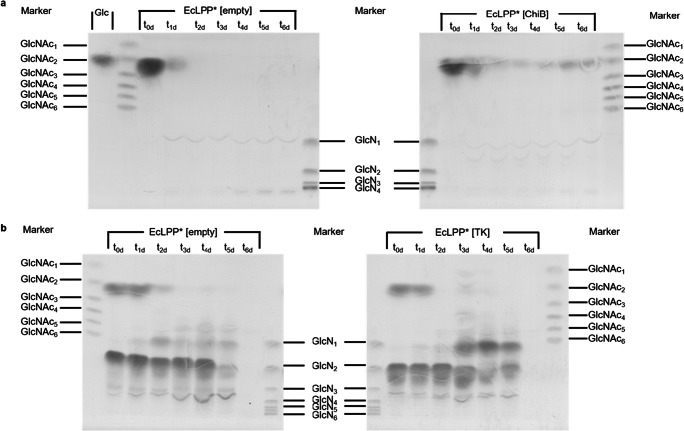
HPTLC analysis of supernatants from cultures of *E. coli* strains EcLPP* [ChiB] (a) and EcLPP* [TK] (b) as well as the empty vector control EcLPP* [empty]. (a) Glc: glucose standard (54 μg), marker: standard GlcNAc-GlcNAc_6_ or GlcN-GlcN_4_ (8 μg each), EcLPP* [empty]: culture supernatant of a culture of EcLPP* [empty] supplied with 0.1% (w/v) colloidal chitin and 20 mM Glc, at time points t0d-t6d. EcLPP* [ChiB]: culture supernatant of a culture of EcLPP* [ChiB] supplied with 0.1% (w/v) colloidal chitin and 20 mM Glc, at time points t0d-t6d. (b) marker: standard GlcNAc-GlcNAc_6_ or GlcN-GlcN_6_ (8 μg each), EcLPP* [empty]: culture supernatant of a culture of EcLPP* [empty] supplied with 12 mM GlcN_2_ and 20 mM Glc, at time points t0-t6. EcLPP* [TK]: culture supernatant of a culture of EcLPP* [TK] supplied with 12 mM GlcN_2_ and 20 mM Glc, at time points t0d-t6d. Application volume of all samples was 15 μl. Identification of Glc, GlcN, GlcN_2_, and GlcNAc_2_ was verified using UHPLC-ESI-MS^n^ (Supplemental Fig. S3)

To test whether the substrate converter can functionally express not only TkCDA but also ChiB and TK, the corresponding genes were introduced separately, yielding two new variants of the substrate converter. These were grown on Glc in the presence of colloidal chitin in the case of EcLPP* [ChiB], and GlcN_2_ in the case of EcLPP* [TK]. Addition of Glc was required as these substrate converters can neither utilize the ChiB-generated GlcNAc_2_ nor the TK-generated GlcN. HPTLC analysis of the culture supernatants showed that Glc was metabolized during the first two days in cultures of EcLPP* [ChiB], and production of GlcNAc_2_ was observed during incubation with colloidal chitin (Fig. 10a), showing that ChiB was functionally expressed. In cultures of EcLPP* [TK], GlcN was formed during incubation with GlcN_2_ (Fig. 10b), showing that TK was functionally expressed, too.

Concomitant expression of all three enzymes (ChiB, TkCDA, and TK) in one substrate-converter strain EcLPP* [ChiB_TK_TkCDA] and its use in co-culture with CgLYS4 on colloidal chitin did not yet result in growth of CgLYS4 (not shown). Therefore, to reduce the metabolic burden due to multiple heterologous protein expression, we tested whether three *E. coli* strains, each expressing only one enzyme, could enable growth of CgLYS4 on colloidal chitin in co-culture (Supplemental Fig. S4). However, also in this approach, no growth of CgLYS4 was detected. In all experiments, a clear decrease of CFUs was seen for the *E. coli* strains, while the CFUs of strain CgLYS4 remained constant.

## Discussion

Establishing a bacterial co-culture based on chitin as a substrate is at the same time highly promising and highly demanding. It is promising not only because chitin is abundantly available from different waste streams, but also because it allows to set up a system in which the substrate converter and producer strains grow on different substrates produced from it, namely GlcN and acetate. It is demanding because chitin is a recalcitrant polymer that forms crystalline fibers embedded in complex matrices such as fungal cell walls, insect cuticles, or crustacean shells, requiring a complex set of enzymes for its degradation (Arnold et al. 2020).

To establish a bacterial co-culture converting chitin to a target product, we first had to introduce different metabolic deficiencies into the substrate converter and the producer strain. Because as an attractive proof of principle, we wanted to establish lysine production from chitin*,* we decided to offer acetate as an energy and carbon source to the substrate converter *E. coli,* and GlcN as an energy, carbon and nitrogen source to the amino acid producer *C. glutamicum*. As a consequence, we had to delete uptake mechanisms for amino sugars in *E. coli,* namely for the monomers GlcNAc (*nagE*) and GlcN (*manXYZ*) as well as for the dimer chitobiose (*chbBCA*) (Plumbridge and Pellegrini 2004; Verma and Mahadevan 2012). We also had to disable *C. glutamicum* from using acetate by deleting genes encoding acetate kinase (*ackA*), phosphotransacetylase (*pta*), CoA-transferase (*cat*), isocitrate lyase (*aceA*), and malate synthase (*aceB*). Additionally, we had to delete the repressor-encoding gene *nanR* to enable *C. glutamicum* to grow on GlcN (Uhde et al. 2013). The producer strain was further improved for performance in the consortium by deleting *ldhA* to prevent cross-feeding of lactate to the substrate converter. Both strategies were successful even though the substrate converter strain EcLPPLYSA* was apparently still able to use GlcN after a longer incubation period. While this was observed in single culture, it is most likely of no concern in co-culture with the producer strain which utilizes GlcN much more efficiently so that it will not be available long enough for *E. coli* to grow on it.

Not unexpectedly, establishing chitin utilization in *E. coli* as the substrate converter proved a lot more demanding than establishing the metabolic deficiencies. Chitin degradation to GlcN and acetate requires the introduction of a whole enzymatic cascade comprised of at least three enzymes as used in this study (Tanaka et al. 2004; Mekasha et al. 2017). In principle, two alternative approaches are feasible. The first step needs to be chitinase-catalyzed depolymerisation of chitin into small chitin oligomers. These can either be deacetylated by a chitin deacetylase yielding acetate and chitosan oligomers which can then be degraded by glucosaminidase to yield GlcN. Alternatively, these last two steps could occur in reverse order, first degrading chitin oligomers using *N*-acetylglucosaminidase into GlcNAc which can then be deacetylated by chitin deacetylase to yield GlcN and acetate. Given that chitin occurs in nature as crystalline fibers embedded into complex biological matrices such as fungal cell walls, insect cuticles, or crustacean shells, even more enzymes such as β-glucanases, proteases and lytic chitin monooxygenases will eventually be required for an efficient utilization of these biomaterials available on large scale from different waste streams. To develop such a system, a bottom-up approach is best suited, step-by-step establishing substrate degradation ‘in reverse’, starting with the final step (Shin et al. 2010; Jia et al. 2016; Gumulya et al. 2018).

Depending on which of the alternative scenarios described above is chosen, the final step would be glucosaminidase-catalyzed degradation of chitosan oligomers to GlcN, or chitin deacetylase-catalyzed degradation of GlcNAc to GlcN and acetate. We opted for the latter scenario as only this one concomitantly produces both substrates required for the growth of the substrate converter and producer strain, allowing to achieve bottom-up proof-of-principle by establishing the co-culture on GlcNAc as a substrate. Consequently, we had to select a suitable chitin deacetylase able to act on the monomer GlcNAc. The only enzyme known with this ability is TkCDA from *T. kodakarensis,* an enzyme naturally involved in chitin utilization by this bacterium (Tanaka et al. 2004). As *T. kodakarensis* is a hyperthermophile, we tested the temperature dependency of the enzyme and found maximum enzyme activity at 54 °C, and about one third lower activity at 37 °C. Interestingly, and unexpectedly given the above scenarios, TkCDA is known, in *T. kodakarensis*, to act in concert with a glucosaminidase, not with a *N*-acetylglucosaminidase. TkCDA can act not only on GlcNAc, but also on chitin oligomers, deacetylating only the GlcNAc unit at the non-reducing end of the oligomers. The thus produced GlcN unit is then cleaved off by glucosaminidase, and the resulting smaller chitin oligomer can again be used as a substrate by TkCDA. For the purpose of establishing the co-culture-based utilization of chitin, this allowed us to start the bottom-up approach using TkCDA-catalyzed deacetylation, leaving both options, i.e. the addition of a glucosaminidase or of an *N*-acetylglucosaminidase, as the next step.

We believe that the chitin deacetylase/glucosaminidase pathway may have evolved in *T. kodakarensis* to avoid the need for a *N*-acetylglucosaminidase which might be toxic for a bacterium with a GlcNAc containing murein-based cell wall. Therefore, an analogous approach was followed when attempting to establish the utilization of chitin polymer by the substrate converter, adding the glucosaminidase TK from the same organism and the chitinase ChiB from *S. marcescens* (Brurberg et al. 1996; Horn et al. 2006). When these enzymes were added separately into the substrate converter EcLPP*, they conveyed the expected abilities, i.e. to cleave chitin into chitobiose and chitobiose into GlcN.

However, when the two enzymes were combined in one strain with a lysine auxotrophy and already expressing TkCDA to generate a substrate converter strain that can be tested in co-culture with lysine producing *C. glutamicum* strain CgLYS4, no growth of the producer strain was observed on colloidal chitin as a substrate. This was also the case when the three enzymes were produced in three separate substrate converter strains and grown in multiple co-culture with the producer strain. Most likely, enzyme production and secretion or enzyme efficiency on colloidal chitin were not sufficient to provide enough substrates for growth of the converter and/or producer strain. Unfortunately, quantification of enzyme activities and of their products as well as of lysine production was not possible due to the complexity of the M9extra medium which interfered with the HPLC analysis of the supernatants. To open up this bottleneck, it will be required to improve secretion even more than already achieved by deleting the *lpp* gene (Shin et al. 2010; Chen et al. 2014; Müller et al. 2016), and to develop a more efficient chitinolytic enzyme machinery, making use of what nature has to offer.

Improving secretion might be achieved by testing different signal peptides for all three enzymes, since it has been shown in literature that the secretion efficiency not only depends on the signal peptide alone, but that it varies depending on the combination of signal peptide and enzyme (Brockmeier et al. 2006; Hemmerich et al. 2016). In addition, other secretion systems could be tested, including heterologous expression of translocation systems (Albiniak et al. 2013) or fusion of the recombinant protein to carrier proteins (Zhang et al. 2006).

Well-known chitin degrading bacteria in soil, marine systems, and freshwater habitats are *S. marcescens* (Vaaje-Kolstad et al. 2013), *Vibrio* spec. (Meibom et al. 2004), and *Aeromonas hydrophila* (Zhang et al. 2017), respectively. All of them possess complex chitinolytic machineries consisting of several chitinases and a chitin-degrading lytic polysaccharide monooxygenase (LPMO). As the co-culture required a freshwater salinity, we investigated the chitinolytic enzymes of *A. hydrophila*. In fact, an *E. coli* strain secreting a chitinase from *A. hydrophila* as well as an LPMO from *S. marcescens* proved that simultaneous production and secretion of these two types of enzymes by *E. coli* is possible (Yang et al. 2017). We have identified the *A. hydrophila* strain AH-1 N based on an enrichment approach with chitin as substrate (Stumpf et al. 2019), and characterized its chitinase AH-1NChi as being rather efficient on crystalline chitin, and as acting synergistically with the LPMO AhLPMO10A from the same strain (Vortmann et al. unpublished). These may in future be used to improve the performance of the substrate converter. Alternatively, additional chitinases with activities complementing that of ChiB such as ChiA and ChiC of *S. marcescens*, alongside its LPMO, might be used (Purushotham et al. 2012; Vaaje-Kolstad et al. 2013; Manjeet et al. 2013).

For all *E. coli* strains that heterologously expressed enzymes, the CFUs declined. A decrease in the number of dividing cells for an *E. coli* strain expressing a recombinant enzyme, measured by their CFU ability, has previously been reported by Andersson et al. (1996). They suggested that cells, whose heterologous gene expression was induced by IPTG, segregate and some cells enter the viable but non-culturable state (VBNC), meaning that they are incapable of division but still retain their metabolic activity. This might be explained by nutrient limitation, as a high amount of energy and carbon is needed for production of the recombinant enzymes and is therefore not available for growth. This may lead to a stress situation for the cells which has been described to lead to the VBNC-status (Oliver 2010; Ramamurthy et al. 2014). As cells which have entered the VBNC-status cannot be detected in CFU-assays, the observed decrease in CFUs of EcLPPLYSA* [TkCDA] does not necessarily imply a decrease in total number of cells in the culture, though this of course can also not be excluded. Clearly though, the amount of secreted TkCDA must have been high enough to provide enough GlcN for growth of CgLYS4 and production of sufficient l-lysine to complement lysine auxotrophy of the substrate converter, even though the CFUs of strain EcLPPLYSA* [TkCDA] decreased. Moreover, growth of the cells in co-culture has only been monitored via CFU counts on solid media, not considering that cells might behave differently in liquid media, possibly even showing growth in liquid medium.

Our study provides proof-of-principle for the bottom-up development of a synthetic bacterial consortium eventually able to utilize the recalcitrant biopolymer chitin from food and biotechnology waste streams for the production of fine chemicals such as amino acids. A number of synthetic consortia have previously been described that divide labour with respect to substrate conversion and product formation, representing steps towards a fully integrated, interdependent mutualistic and non-competitive consortium as demonstrated here for the first time (Sgobba and Wendisch 2020). Conversion of cellulose to isobutanol has been demonstrated by co-culturing the cellulase-secreting fungus *Trichoderma reesei* with an isobutanol producing *E. coli* strain (Xin et al. 2019) and similarly, conversion of sugarcane bagasse slurry to ethanol has been achieved by co-culturing *Saccharomyces cerevisiae* that ferments glucose to ethanol and glucose negative ethanologenic *E. coli* that ferments xylose to ethanol (Wang et al. 2019). A co-culture of an l-lysine auxotrophic, naturally sucrose-negative *E. coli* strain and a *C. glutamicum* strain producing l-lysine and fructose from sucrose established commensalism in which the *E. coli* strain benefitted from the *C. glutamicum* strain that, however, was not dependent on the *E. coli* strain so that no mutualistic interdependence was established (Sgobba et al. 2018). An extension of this consortium comprised an α-amylase secreting l-lysine auxotrophic *E. coli* strain, allowing it to mutualistically grow on starch with a naturally amylase-negative lysine producing *C. glutamicum* strain (Sgobba et al. 2018). Growth of this mutualistic consortium required lysine cross-feeding and hydrolysis of starch to glucose, for which both strains competed as carbon and energy source for growth. The GlcNAc-converting consortium described here is equally mutualistic and depending on lysine cross-feeding, but it extends the concept significantly by avoiding competition: here, the carbon source is divided between the partners such that *E. coli* grows with acetate and *C. glutamicum* with GlcN. Stepwise extension of the concept of division of labour regarding access to substrates will likely develop further as has been seen with respect to division of labour between different steps of product formation from shorter to longer linear cascades to converging designs (Sgobba and Wendisch 2020).

Eventually, this concept of labour division within a fully integrated, interdependent, mutualistic, non-competitive synthetic microbial consortium can be developed into a versatile platform for modular synthetic biotechnology where substrate converter strains using different substrates will be combined with producer strains yielding different products. Clearly, the benefit of using hexosamines or aminosugar containing polymers such as chitin as a substrate is that in addition to providing a carbon source, these carbohydrates also serve as a source of nitrogen which is required in high amounts for the production of many interesting organic compounds, such as amino acids. Moreover, unlike widely used carbon sources such as glucose or starch, chitin cannot be used as food, feed, or fuel, avoiding competition with these fields.

## Supplementary Information

ESM 1 (PDF 383 kb)

## Data Availability

Data and material described in this study are available from the authors upon reasonable request and availability.
