# Supplementary material for: A bottom-up approach towards a bacterial consortium for the biotechnological conversion of chitin to l-lysine
Source: Appl Microbiol Biotechnol. 2021 Feb 1;105(4):1547–61. doi: 10.1007/s00253-021-11112-5 (PMC7880967; doi:10.1007/s00253-021-11112-5)
Supplement: Supplementary file 1 — (PDF 383 kb) [file 253_2021_11112_MOESM1_ESM.pdf]

Supplementary material to **Applied Microbiology and Biotechnology**

A bottom-up approach towards a bacterial consortium for the biotechnological conversion of chitin to L-lysine

Marina Vortmann[1‡], Anna K. Stumpf[2‡], Elvira Sgobba[3,#], Mareike E. Dirks-Hofmeister[4], Martin Krehenbrink[5], Volker F. Wendisch[3], Bodo Philipp[2], and Bruno M. Moerschbacher[1*]

[1] Institute for Biology and Biotechnology of Plants, University of Münster, Schlossplatz 8, 48143 Münster, Germany

[2] Institute for Molecular Microbiology and Biotechnology, University of Münster, Correnssstr. 3, 48149 Münster, Germany

[3] Chair of Genetics of Prokaryotes, Faculty of Biology & CeBiTec, University of Bielefeld, P.O. Box 100131, 33501 Bielefeld, Germany

[4] WeissBioTech GmbH, An der Hansalinie 48-50, 59387 Ascheberg, Germany

[5] Cysal GmbH, Mendelstraße 11, 48149 Münster, Germany

[‡] these authors contributed equally to this work

[#] current address: Department of Forest Genetics and Plant Physiology, SLU, Skogsmarksgränd 17, 90183 Umeå, Sweden

*Corresponding author:

Bruno M. Moerschbacher, Institute for Biology and Biotechnology of Plants, University of Muenster, Schlossplatz 8, 48143 Münster, Germany

e-mail address: moersch@uni-muenster.de

phone: +49 251 8324794, fax: +49 251 8328371

Table S1a Strains used in this study

Escherichia coli		
**Abbreviation**	**Relevant characteristics**	**Reference**
EcWT	*E. coli* W3110, F- lambda- IN*(rrnD-rrnE)1 rph-1*	(Bachmann 1972)
EcNagE*	EcWT with *ΔnagE ΔmanXYZ*	This work
EcCHB	*E. coli* W3110 with *ΔnagE ΔmanXYZ ΔchbBCA::CM*, Cm^R	This work
EcCHB*	EcCHB; chloramphenicol resistance gene removed	This work
EcLPP	EcCHB with *Δlpp::CM;* Cm^R	This work
EcLPP*	EcLPP; chloramphenicol resistance gene removed	This work
EcLPPLYSA	EcLPP* with *ΔlysA::CM;* Cm^R	This work
EcLPPLYSA*	EcLPPLYSA; chloramphenicol resistance gene removed	This work
EcLPP* [empty]	EcLPP* with [pPRII+ (empty-vector)], Cm^R	This work
EcLPP* [TkCDA]	EcLPP* with [pPRII+::*C.vio*-TkCDA-StrepII], Cm^R	This work
EcLPPLYSA* [TkCDA]	EcLPPLYSA* with [pPRII+::*C.vio*-TkCDA-StrepII], Cm^R	This work
EcLPP* [TK]	EcLPP* with [pPRII+::*pelB*-TK-StrepII], Cm^R	This work
EcLPP* [ChiB]	EcLPP* with [pPRII+::*pelB*-ChiB-StrepII], Cm^R	This work
EcNagE* [TkCDA]	EcNagE* [pPRII+::*C.vio*-TkCDA-StrepII], Cm^R	This work
EcLPP* [ChiB_TK_TkCDA]	EcLPP* with [pPRII+::*pelB*-ChiB-StrepII_*pelB*-TK-StrepII_*pelB*-TkCDA-StrepII], Cm^R	This work
Corynebacterium glutamicum		
**Abbreviation**	**Relevant characteristics**	**Reference**
DM1729	*C. glutamicum* ATCC 13032 carrying chromosomal mutations *pycP458S, homV59A, lysCT311I*	(Georgi et al. 2005)
CgLYS4	*C. glutamicum* DM1729 *Δpta-ackA Δcat ΔldhA ΔaceAB ΔnanR*	(Sgobba et al. 2018)

Cm^R: chloramphenicol resistance

Table S1b Plasmids used in this study

Plasmids		
**Name**	**Relevant characteristics**	**Reference**
pPRII+::pelB-TK-StrepII	pPRII+ with pelB signal peptide, TK (glucosaminidase) from *Thermococcus kodakarensis* KOD1 and StrepII-Tag	This work
pPRII+::pelB-ChiB-StrepII	pPRII+ with pelB signal peptide, ChiB (chitinase) from *Serratia marcescens* and StrepII-Tag	This work
pPRII+::C.vio-TkCDA-StrepII	pPRII+ with sequence for *C. violaceum* signal peptide, TkCDA (chitin deacetylase) from *Thermococcus kodakarensis* KOD1 and StrepII-Tag	This work
pPRII+::pelB-ChiB-StrepII_pelB-TK-StrepII_pelB-TkCDA-StrepII	pPRII+ ChiB, TK and TkCDA; all enzymes include a pelB signal peptide and a StrepII-tag	This work
pPRII+ (empty vector)	pPRII+ empty-vector control	EP2848691A1 (patent)

Table S2 Oligonucleotides used in this study

No.	Name	Oligonucleotide sequence (5'-3')	Description
1	nagE_fow	**AAAAATACGGCTTTAAACGAGCCAA ATAGGGTTCTCGTAGGGGGAATAAG** GTGTAGGCTGGAGCTGCTTC	Amplification of Cm^R from pKD3 for disruption of *nagE*
2	nagE_rev	**TTGTCATTGTTGGATGCGACGCTCA AGCGTCGCATCAGGCATAAAGCAGA** CATATGAATATCCTCCTTAG	Amplification of Cm^R from pKD3 for disruption of *nagE*
C1	nagE_contr_fw	TATACATTTGCCGACCTG TTCAGC	Control of *nagE* deletion
C2	nagE_contr_rev	TATATGCACTGGCCTTTA TAGTCC	Control of *nagE* deletion
3	manXYZ_fow	**AAAATACATCTGGCACGTTGAGGTG TTAACGATAATAAAGGAGGTAGCAA** GTGTAGGCTGGAGCTGCTTC	Amplification of Cm^R from pKD3 for disruption of *manXYZ*
4	manXYZ_rev	**CCTCCAGATAAAAAAACGGGGCCAA AAGGCCCCGGTAGTGTACAACAGTC** CATATGAATATCCTCCTTAG	Amplification of Cm^R from pKD3 for disruption of *manXYZ*
C3	manXYZ_contr_fw	CGATTCGATTGTGGACGACG	Control of *manXYZ* deletion
C4	manXYZ_contr_rev	ACCAGGTCCGTGATTGTCAT	Control of *manXYZ* deletion
5	chbBCA_fow	**AGGCTTGCGGAGTGTCTGGCTGACA GATAATCGTCGATGAGGGCAGTTTT** GTGTAGGCTGGAGCTGCTTC	Amplification of Cm^R from pKD3 for disruption of *chbBCA*
6	chbBCA_rev	**GGGCAGTGGCAATTTCCGGCGCGTT AATCACTGGCTGCATCATCGACTCC** CATATGAATATCCTCCTTAG	Amplification of Cm^R from pKD3 for disruption of *chbBCA*
C5	chbBCA_contr_fw	ATCTTCGCGAATTATTTGCCC	Control of *chbBCA* deletion
C6	chbBCA_contr_rev	ATTTCCGGCGCGTTAATCAC	Control of *chbBCA* deletion
7	lysA_fow	**CTTTTTATGA TGTGGCGTAA TCATAAAAAA GCACTTATCT GGAGTTTGTT**GTGTAGGCTGGAGCTG CTTC	Amplification of Cm^R from pKD3 for disruption of *lysA*
8	lysA_rev	**CAACTCCGTCGCTGGAGGCAAGTCA TCATGCAACCAGCGACTAACCGCAG** CATATGAATATCCTCCTTAG	Amplification of Cm^R from pKD3 for disruption of *lysA*
C7	lysA_contr_fw	CAAACAGACGCAGTCCTTGC	Control of *lysA* deletion
C8	lysA_contr_rev	AGTGGTATTGCGGCCAATGA	Control of *lysA* deletion
9	lpp_fow	**AATACTTGTAACGCTACATGGAGAT TAACTCAATCTAGAGGGTATTAATA** GTGTAGGCTGGAGCTGCTTC	Amplification of Cm^R from pKD3 for disruption of *lpp*

10	lpp_rev	**ACAAAAAAAATGGCGCACAATGTGCGCCATTTTTCACTTCACAGGTACTA**CATATGAATATCCTCCTTAG	Amplification of Cm^R from pKD3 for disruption of *lpp*
C9	lpp_contr_fw	GTAACGCTACATGGAGATTAAC	Control of *lpp* deletion
C10	Lpp_contr_rev	GACGCAGTAGCGGTAAACGGCAG	Control of *lpp* deletion

Underlined letters restriction site

Table S3 Sequences of primers used for construction of vectors

Name	Oligonucleotide sequence (5'-3')	Description
pPRII+::chiB_for	ATGACTCGAGCTGCAGAAGC	Excision of *TK* and *TkCDA* from pPRII+::chiB_TK_TkCDA to construct pPRII+::pelB-chiB-StrepII
pPRII+::chiB_for	CTAGATTATTTTTCAAATTGCGGGTGGC	Excision of *TK* and *TkCDA* from pPRII+::chiB_TK_TkCDA to construct pPRII+::pelB-chiB-StrepII
pPRII+::TkCDA_for	ATGAAATACCTGCTGCCG	Excision of *chiB* and *TK* from pPRII+::chiB_TK_TkCDA to construct pPRII+::pelB-TkCDA-StrepII
pPRII+::TkCDA_rev	TTGGAATTCTGTTTCCTGTG	Excision of *chiB* and *TK* from pPRII+::chiB_TK_TkCDA to construct pPRII+::pelB-TkCDA-StrepII
C.vio-TkCDA_for	*ATGTTGTTAGCATTGGGACAACATGCTTGGGCTGCTGCA*ATGGTGTTCGAAGAATTTAACAACTTTG	Exchange of pelB-leader for *C. violaceum*-Tag
C.vio-TkCDA_rev	*GGCCATTGCGATAGCACGACCTGTAGTGCGGCGCAT*TTGGAATTCTGTTTCCTGTGTGAAATTG	Exchange of pelB-leader for *C. violaceum*-Tag
TK_GA_for	**CACAGGAAACAGAATTCCAA**ATGAAATACCTGCTGCCG	Amplification of TK from pPRII+::chiB_TK_TkCDA
TK_GA_rev	GCTTCTGCAGCTCGAGTCATTTTTCA**AATTGCGGGTGAG**	Amplification of TK from pPRII+::chiB_TK_TkCDA

Italic letters: *C. violaceum*-Tag; Bold letters: Overlapping region

Table S4 Construction of plasmids

Plasmid	Template	Primers	Cloning method
**pPRII+::pelB-chiB-StrepII_pelB-TK-StrepII_pelB-TkCDA-StrepII**	pPRII::Syn_OP	-	Digestion with *Eco*RV, religation of vector
pPRII+::pelB-chiB-StrepII	pPRII+::pelB-chiB-StrepII_pelB-TK-StrepII_pelB-TkCDA-STrepII	pPRII+::chiB_for, pPRII+::chiB_rev	Back-to-back PCR, religation
pPRII+::pelB-TkCDA-StrepII	pPRII+::pelB-chiB-StrepII_pelB-TK-StrepII_pelB-TkCDA-StrepII	pPRII+::TkCDA_for, pPRII+::TkCDA_rev	Back-to-back PCR, religation
pPRII+::pelB-TK-StrepII	pPRII+::pelB-TkCDA-StrepII (vector backbone)	pPRII+::chiB_for and pPRII+::TkCDA_rev for vector backbone	Gibson assembly using Gibson Assembly® Master Mix (NEB, Ipswich, MA, USA)
	pPRII+::pelB-chiB-StrepII_pelB-TK-StrepII_pelB-TkCDA-StrepII (insert)	TK_GA_for and TK_GA_rev for insert	
**pPRII+::*C.vio*-TkCDA-StrepII**	pPRII+::pelB-TkCDA-StrepII	*C.vio*-TkCDA_for, *C.vio*-TkCDA_rev	Back-to-back PCR, religation

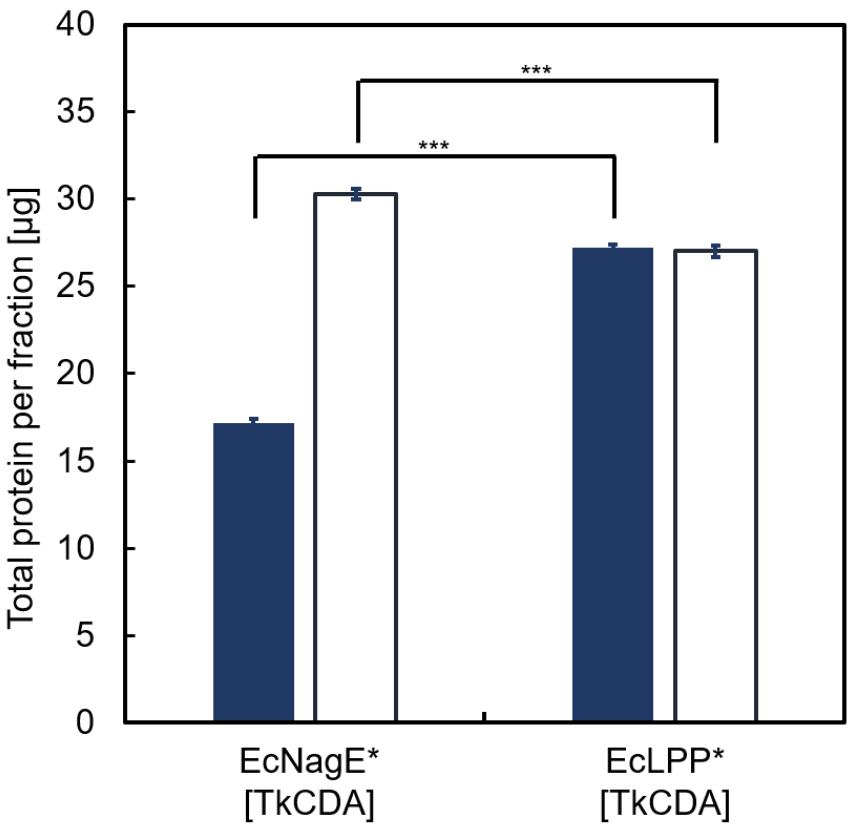

Fig. S1 Secretion of chitin deacetylase TkCDA. Amount of chitin deacetylase TkCDA in the extracellular medium (filled bars) and in the cellular fraction (open bars) of EcLPP* [TkCDA] and *E. coli* W3110 *ΔnagE ΔmanXYZ* [pPRII::*C.vio*-TkCDA-StrepII] (EcNagE* [TkCDA]). Error bars indicate standard deviation (n = 3). Statistically significant difference at *** $P < 0.001$.

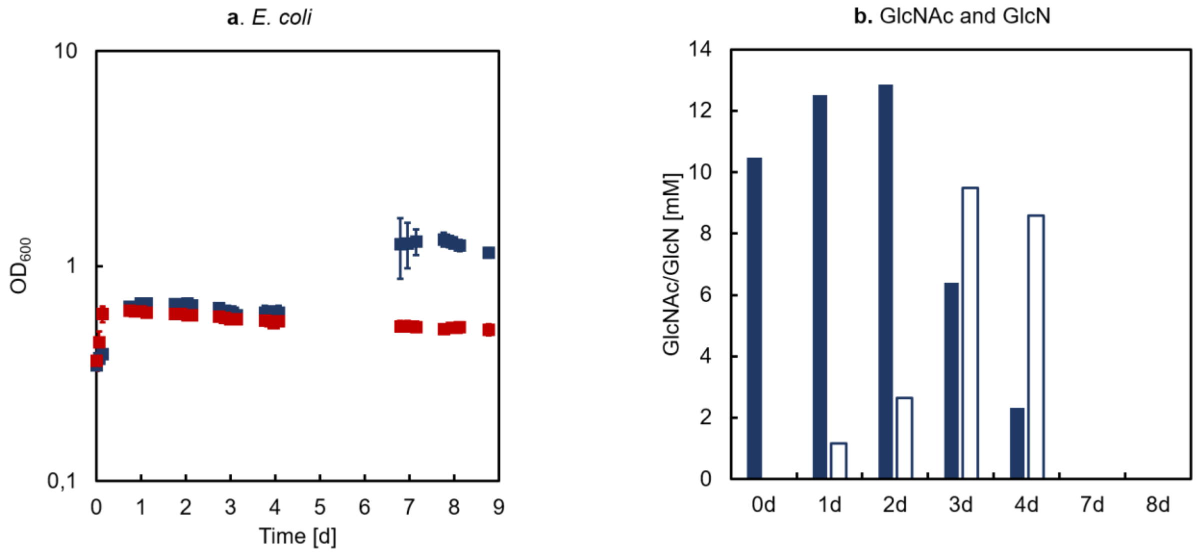

Fig. S2 Growth of *E. coli* strains on GlcNAc and quantification of metabolites. (a) Optical density (OD_{600}) of the strain EcLPP* [empty] (red squares and dashed line) and EcLPP* [TkCDA] (blue squares and solid line) cultivated with 20 mM *N*-acetylglucosamine (GlcNAc) as sole carbon and energy source. *E. coli* cells were induced with 0.2 mM IPTG at t_0. Error bars indicate standard deviation (n = 3). (b) Concentration of GlcNAc (filled bars) and glucosamine (open bars) in the supernatant of EcLPP* [TkCDA] measured using UHPLC-ELSD-ESI-MS^n as described in Stumpf et al. 2019.

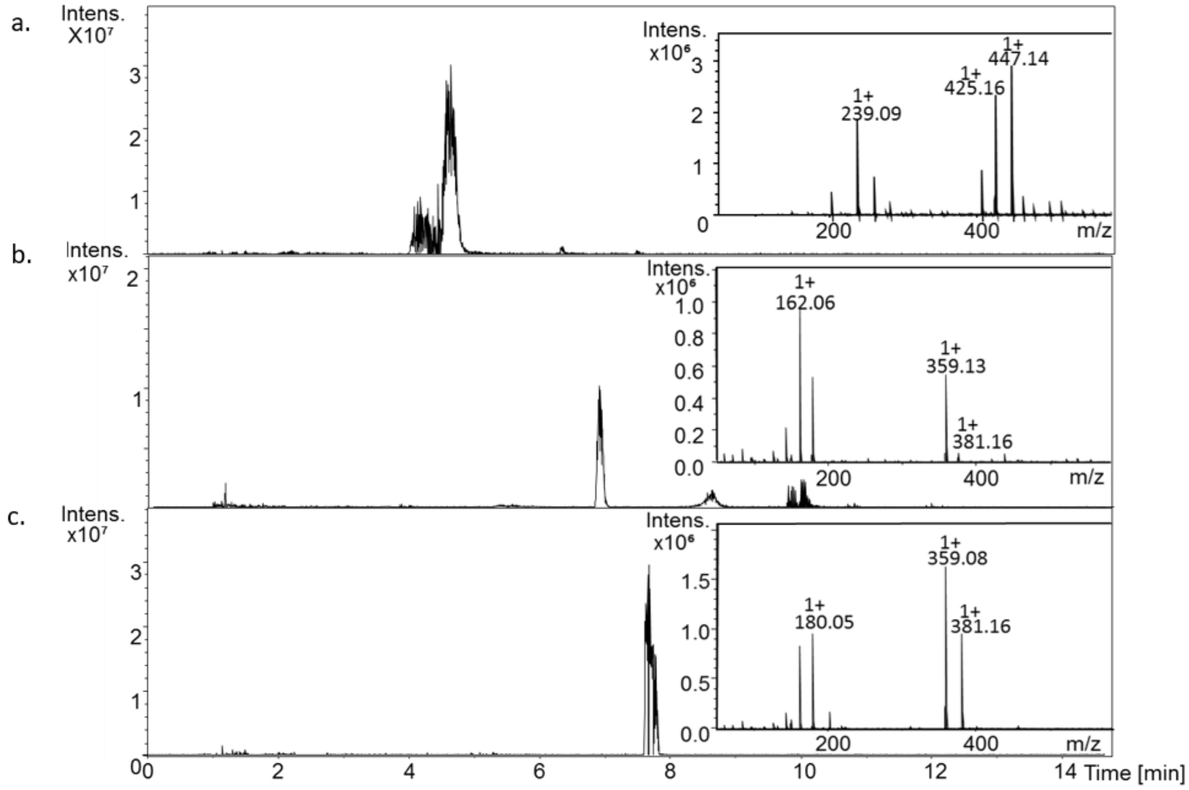

Fig. S3 Extracted ion chromatograms (EICs) of UHPLC-ESI-MS analysis of culture supernatants of substrate converters expressing ChiB, TK, or TkCDA cultured in the presence of their respective substrates (TkCDA: GlcNAc, TK: $GlcN_2$, ChiB: colloidal chitin) at time point t3 (72 h). Inserts show the mass to charge ratio of the respective peaks. A) EIC of mass 425.18 ($GlcNAc_2$, H+ adduct): EcLPP* [ChiB] cultured in M9 minimal medium with 20 mM glucose as carbon source and 0.1% (wt/vol) colloidal chitin. B) EIC of mass 359.17 (two GlcN units, H+ adduct): EcLPP* [TK] cultured in M9 minimal medium with 20 mM glucose as carbon source and 12 mM $GlcN_2$. C) EIC of mass 359.17 (two GlcN units, H^+ adduct): EcLPP* [TkCDA] cultured in M9 minimal medium with 40 mM GlcNAc, no additional carbon source.

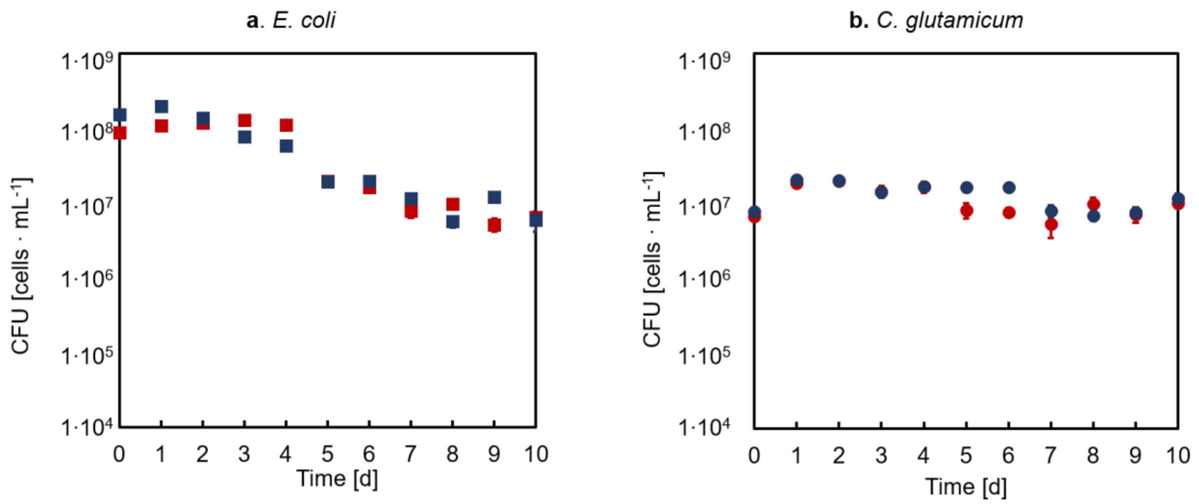

Fig. S4 Cultivation of synthetic microbial consortia on colloidal chitin. Cultivation of the co-culture of EcLPP* [ChiB], EcLPP* [TK], and ECLPP* [TkCDA] together with CgLYS4 and co-culture of EcLPP* [empty-vector] and CgLYS4 on 0.5% colloidal chitin supplemented with 5 mM acetate as sole carbon and energy sources. *E. coli* cells harbouring a plasmid were induced with 0.2 mM IPTG at t_0. (a) CFUs of strains EcLPP* [ChiB], EcLPP* [TK], and ECLPP [TkCDA] (blue squares and solid line) and EcLPP* [empty-vector] (red squares and dashed line). (b) CFUs of strain CgLYS4 in co-culture with EcLPP* [ChiB], EcLPP* [TK], and ECLPP* [TkCDA] (blue dots and solid line) and in co-culture with EcLPP* [empty-vector] (red dots and dashed line). Error bars indicate standard error of the mean (n = 3).

References

Bachmann BJ (1972) Pedigrees of some mutant strains of *Escherichia coli* K-12. Bacteriol Rev 36:525–57

Georgi T, Rittmann D, Wendisch VF (2005) Lysine and glutamate production by *Corynebacterium glutamicum* on glucose, fructose and sucrose: Roles of malic enzyme and fructose-1,6-bisphosphatase. Metab Eng 7:291–301 . https://doi.org/10.1016/j.ymben.2005.05.001

Sgobba E, Stumpf AK, Vortmann M, Jagmann N, Krehenbrink M, Dirks-Hofmeister ME, Moerschbacher B, Philipp B, Wendisch VF (2018) Synthetic *Escherichia coli-Corynebacterium glutamicum* consortia for L-lysine production from starch and sucrose. Bioresour Technol 260:302–310 . https://doi.org/10.1016/j.biortech.2018.03.113

Stumpf AK, Vortmann M, Dirks-Hofmeister ME, Moerschbacher BM, Philipp B (2019) Identification of a novel chitinase from *Aeromonas hydrophila* AH-1N for the degradation of chitin within fungal mycelium. FEMS Microbiol Lett 366:1–9 . https://doi.org/10.1093/femsle/fny294
